# Supplementary material for: Development of a computer-aided design software for the quantitative evaluation of aesthetic damage
Source: PLoS One. 2019 Dec 18;14(12):e0226322. doi: 10.1371/journal.pone.0226322 (PMC6919621; doi:10.1371/journal.pone.0226322)
Supplement: S1 Code site — 1. (ZIP) [file pone.0226322.s001.zip › [EESC_jr][Projeto]Relatorio_07_21/reports/templates_zip/formwizard-master.zip!/formwizard-master/examples/example_16_have_the_wizard_ignore_specific_inputs_during_transition.html]

xml version="1.0" encoding="UTF-8"?


JQuery Form Wizard


### Example of how to make the wizard ignore some input fields by using the class 'wizard-ignore'.

- Wizard with two steps
- On the first step the branching is decided depending on which country is selected.
- The input with id:s man and woman have the class 'wizard-ignore' and are not touched by the wizard during step transitions
- The inputs with id:s man and woman are enabled and disabled outside of the wizard's control (on the change event of the select box with id 'sex')

---


First step - Country  
Country  


Sweden
Finland
  
Sex  


man
woman
  

shown if man is selected

Social Security Number  

This is shown if woman was selected

Social Security Number  


 -


---
